# DNA Methylation Analysis of *BRD1* Promoter Regions and the Schizophrenia rs138880 Risk Allele

**DOI:** 10.1371/journal.pone.0170121

**Published:** 2017-01-17

**Authors:** Mads Dyrvig, Per Qvist, Jacek Lichota, Knud Larsen, Mette Nyegaard, Anders D. Børglum, Jane H. Christensen

**Affiliations:** 1 Department of Biomedicine, Aarhus University, Aarhus, Denmark; 2 Lundbeck Foundation Initiative for Integrative Psychiatric Research, iPSYCH, Aarhus University, Aarhus, Denmark; 3 Department of Health Science and Technology, Aalborg University, Aalborg, Denmark; 4 Centre for Integrative Sequencing, iSEQ, Aarhus University, Aarhus, Denmark; 5 Department of Molecular Biology and Genetics, Aarhus University, Aarhus, Denmark; Universita degli Studi di Napoli Federico II, ITALY

## Abstract

The bromodomain containing 1 gene, *BRD1* is essential for embryogenesis and CNS development. It encodes a protein that participates in histone modifying complexes and thereby regulates the expression of a large number of genes. Genetic variants in the *BRD1* locus show association with schizophrenia and bipolar disorder and risk alleles in the promoter region correlate with reduced *BRD1* expression. Insights into the transcriptional regulation of *BRD1* and the pathogenic mechanisms associated with *BRD1* risk variants, however, remain sparse. By studying transcripts in human HeLa and SH-SY5Y cells we provide evidence for differences in relative expression of *BRD1* transcripts with three alternative 5’ UTRs (exon 1C, 1B, and 1A). We further show that expression of these transcript variants covaries negatively with DNA methylation proportions in their upstream promoter regions suggesting that promoter usage might be regulated by DNA methylation. In line with findings that the risk allele of the rs138880 SNP in the *BRD1* promoter region correlates with reduced *BRD1* expression, we find that it is also associated with moderate regional *BRD1* promoter hypermethylation in both adipose tissue and blood. Importantly, we demonstrate by inspecting available DNA methylation and expression data that these regions undergo changes in methylation during fetal brain development and that differences in their methylation proportions in fetal compared to postnatal frontal cortex correlate significantly with *BRD1* expression. These findings suggest that *BRD1* may be dysregulated in both the developing and mature brain of risk allele carriers. Finally, we demonstrate that commonly used mood stabilizers Lithium, Valproate, and Carbamazepine affect the expression of *BRD1* in SH-SY5Y cells. Altogether this study indicates a link between genetic risk and epigenetic dysregulation of *BRD1* which raises interesting perspectives for targeting the mechanisms pharmacologically.

## Introduction

*BRD1* encodes the bromodomain-containing protein 1 (BRD1), which is widely expressed in human tissues including the brain [1]. BRD1 has been identified in protein complexes possessing acetyltransferase activity towards histone H3 [2,3] and it binds chromatin in regions adjacent to transcription start sites (TSSs) of numerous genes [3,4]. Inactivation of *Brd1* in mice is incompatible with postnatal life due to severe embryonic mal-development including impaired neural tube closure [3]. Cerebral changes in *Brd1* expression upon electroconvulsive seizures [5] and chronic restraint stress in rats [6], suggest an important function of BRD1 in the mature CNS that might include a role in gene regulatory processes underlying adaptation to stress.

The *BRD1* promoter SNP, rs138880, was recently identified as the variant showing the most significant association with schizophrenia in a large GWAS meta-analysis (>11,000 cases and >10,000 controls) ensued by a family-based replication study (>6,000 individuals including >3,000 cases) [7]. This augmented previous studies linking rs138880 with both schizophrenia and bipolar disorders in large Caucasian case-control samples [8,9] as well as the identification of a susceptibility locus containing *BRD1* in the Faroese population [10]. The *BRD1* locus approached genome-wide significance in the Psychiatric Genomics Consortium (PGC) schizophrenia mega-GWASs using conventional statistical methods (*p* = 4.38E-05 in PGC1 [11] and *p* = 3.31E-07 in PGC2 [12], and moreover it was found genome-wide significant and predicted to be highly replicable when applying an Empirical Bayes statistical approach already in the smaller PGC1 data set [13].

We have recently provided evidence that individuals carrying the schizophrenia risk allele (the C-allele) of rs138880 (or SNP alleles in high LD with it) express significantly less *BRD1* mRNA than non-carriers both in blood and brain [14] suggesting that the promoter variant has a role in transcriptional regulation of *BRD1*. That this effect can be attributed either fully or partly to the promoter risk allele of rs138880 is substantiated by our *in vitro* evidence for a lower transcriptional drive of the C allele compared to the A allele in a dual luciferase promoter assay using mouse neuroblastoma Neuro2A cells [14]. In addition, we show that reduced expression of *Brd1* in a genetically modified mouse strain is associated with several phenotypes with translational relevance to schizophrenia and these phenotypes are co-occurring with brain region expression changes affecting schizophrenia risk genes to a higher degree than expected by change [14].

The majority of risk variants associated with schizophrenia do not directly alter protein sequence but rather seem to be important for regulating gene expression [12,15] being in line with the more general findings that disease-associated variants affect transcription factor recognition sequences and frequently alter allelic chromatin states [16]. In human brain samples, it has been established that methylation of a high number of CpG sites show significant cis associations with SNPs and a lower number show significant trans associations [17]. A recent study has demonstrated that 62 out of the 104 (59.6%) genome-wide significant loci in schizophrenia investigated in the study had a risk (or proxy) SNP that is a methylation quantitative-trait locus (metQTL) in human cortex [18].

To reveal insights into the transcriptional regulation of *BRD1* and the dysfunction associated with the rs138880 risk allele of the *BRD1* promoter, we have delineated *BRD1* promoter usage and DNA methylation patterns in human cell lines and tissue. We have further studied the effect of three different mood stabilizers on *BRD1* expression and promoter DNA methylation.

## Materials and Methods

### Cell culture

Human adenocarcinoma HeLa cells (American Type Culture Collection, CCL-2) and human neuroblastoma SH-SY5Y cells (American Type Culture Collection, CRL-2266) were cultured in RPMI 1640 Medium, GlutaMAX^™^ Supplement containing 10% FCS and 1% Penicillin-Streptomycin (10,000 U/mL) (all from Life Technologies, CA, USA). For bisulfite sequencing experiments cells were harvested during these standard conditions. For the experiments involving drugs we used Zebularine (Z4775), Valproic acid sodium salt (P4543), Lithium chloride (L7026), and Carbamazepine (C4024) (all from Sigma, MO, USA). Zebularine and Carbamazepine were dissolved in DMSO. Lithium and Valproate were dissolved in EtOH. Drugs were diluted in culture medium to give the minimum and maximum therapeutic concentrations: Valproate, 0.3 and 0.6 mM; Lithium, 0.6 and 1.2 mM; Carbamazepine 0.05 and 0.1 mM [19]. Zebularine was diluted in culture medium to doses of 0.05, 0.1, and 0.25 mM. In all experiments, control cells were exposed to the same concentration of solvent (DMSO or EtOH) but without addition of drug. All experiments were performed in triplicates. On day one, 2,000 HeLa cells/cm^2^ or 20,000 SH-SY5Y cells/cm^2^ were plated in 6-well plates (Techno Plastic Products, Trasadingen, Switzerland) and left for 24 hours to adhere. On day two, the drugs were added directly to the culture medium at the indicated concentrations and left for 24 hours. On day three, the cell medium was replaced with fresh medium containing drug at the indicated concentrations and left for 48 hours. On day 5, DNA and RNA were extracted with an AllPrep DNA/RNA Mini Kit (Qiagen, Hilden, Germany) according to manufacturer’s protocol.

### Subjects

The subjects used for DNA methylation analysis included 48 female Caucasian university students. DNA was isolated from blood and the subjects had previously been genotyped for SNP rs138880 as described in [9]. A total of 24 homozygous (A/A) and 24 heterozygous (A/C) individuals were included. Subjects provided written, informed consent for participation, and approval was obtained from the ethics committee for Central Denmark Region.

### RNA quality assessment and cDNA synthesis

RNA concentration and purity was assessed using a NanoDrop Spectrophotometer (Thermo Scientific, Wilmington, DE, USA). Integrity of the RNA was assessed on a 1.2% agarose gel stained with ethidium bromide and cDNA synthesis was performed as described [20]. For cells grown with Zebularine, 400 ng of total RNA was used and for the remaining drugs, 1 μg of total RNA was reverse transcribed. Detailed description of the materials and methods used for transcript variant analysis can be found in S1 Supporting Information and related primer sequences, expected amplicon sizes, and PCR extension times are listed in S1 Table.

### Real-time quantitative PCR

The real-time quantitative PCR (qPCR) reactions were run in a LightCycler^®^480 System (Roche Applied Science, Mannheim, Germany), using white 384-well PCR plates sealed with adhesive sealing film (Roche Applied Science). The reaction mixtures consisted of 1x LightCycler^®^ 480 SYBR Green I Master (Roche Applied Science), 0.5 μM of each primer, and 2.5 μL diluted cDNA in a total volume of 5 μL. The cycling protocol started with one cycle of 95°C for 10 min, followed by 45 cycles of: 95°C for 10 sec, 60°C for 20 sec, and 72°C for 20 sec. Melting curve analyses were performed subsequent to amplification of target sequences. All samples were run in triplicate. Primer sequences are listed in S2 Table. For measuring the basal expression levels of total *BRD1* and transcript variants containing exon 1C, 1B, and 1A in HeLa and SH-SY5Y cells, gene expression was calculated by the efficiency corrected method [21] using the expression of the *TBP* and *PGK1* genes for normalization. For experiments involving drug addition to cell cultures, relative gene expression was calculated for each gene by the “standard curve method” [22] using serial dilutions of 1/5, 1/25, 1/125, and 1/625 from a pool of cDNA. Five normalization candidate genes (*HPRT*, *PGK1*, *POLR2A*, *RPS13*, and *TBP*) were included for each treatment and target gene expression was normalized to the geometric mean of the expression levels of the two most stable reference genes as determined by the Normfinder software [23]. The efficiencies of all primers pairs designed were tested prior to final sample measurements and only those with efficiencies close to 100% (90–110%) were included in the study.

### Bisulfite conversion

500 ng DNA isolated from each subject were bisulfite converted using an EZ DNA Methylation kit (Zymo Research, CA, USA) according to the manufacturer’s instructions. Briefly, after addition of M-dilution buffer to DNA each sample was incubated at 37°C for 15 min. CT conversion reagent was added and samples were incubated at 50°C for 16 hours before final clean up. The same procedure was used for conversion of DNA extracted from cell lines except for using 400 ng DNA of each sample.

### Bisulfite sequencing of single clones

Four genomic regions upstream of the *BRD1* gene were included for bisulfite sequencing. The regions were amplified with HotStarTaq DNA Polymerase (Qiagen) with the primers listed in S3 Table. The primers were designed to be methylation independent by avoiding CpGs at primer binding sites. To prevent amplification bias, the primers were tested at several annealing temperatures, and the highest temperature providing sufficient product was selected [24]. For each region, a 20 μL reaction was prepared according to standard protocol with 30 ng bisulfite converted DNA (theoretical amount), and a final concentration of 0.5 μM of each primer, and 1.5 mM MgCl_2_. The cycling conditions were: 95°C for 15 min followed by 40 cycles of: 94°C 30 sec, x°C 2 min, and 72°C 1 min, with a final step at 72°C for 10 min. The annealing temperature for each assay is listed in S3 Table. PCR products were separated on a 2% agarose gel stained with ethidium bromide. PCR products of expected sizes were cut out from the gel and isolated with a QIAquick Gel Extraction Kit (Qiagen). PCR products were cloned using the InsTAclone^™^ PCR Cloning Kit (Thermo Scientific) and selected plasmid DNA was extracted with a GeneJET Plasmid Miniprep Kit (Thermo Scientific). Plasmid was sequenced with primers M13-F20 and if necessary T7-981079 (GATC Biotech, Constance, Germany).

### Pyrosequencing assay

Two genomic regions upstream of the *BRD1* gene were selected for pyrosequencing and amplified with the primers listed in S3 Table. The first assay (Pyrosequencing region 2) was designed to target rs138880 and cg15145965 (specifying a probe for a specific CpG site on the Illumina Infinium Human Methylation 450K Bead Array). The second assay (Pyrosequencing region 3) was designed to target cg06057569 and CpG sites in its close proximity. As described above, the primers were designed to be methylation independent and the highest possible annealing temperature was used. Amplification was performed as described above with the following deviations: 50 μL reactions were prepared with 40 ng bisulfite converted DNA (theoretical amount) and a final concentration of 0.2 μM of each primer was used. The annealing temperature for each assay is listed in S3 Table. Amplification products were sequenced on a PyroMark Q24 Advanced (Qiagen) using the PyroMark Q24 Advanced CpG Reagents (Qiagen), according to the manufacturer’s instructions.

### MetQTL analysis and correlation testing

Associations between DNA methylation proportions and rs138880 genotype (cis-MetQTLs ± 5 kb from SNP) in adipose tissue was analyzed using the Genevar (GENe Expression VARiation) platform [25] applying default settings with a *p*-value cutoff of <0.001. The samples (n = 603) were collected from the TwinsUK cohort comprising healthy females of European descent [26]. For details on statistics see Grundberg et al. [27]. Correlation between methylation proportion and fetal age was assessed with the Pearson’s correlation test (assuming that both X and Y values were sampled from populations that follow a Gaussian distribution) using the GraphPad Prism 5.0 software.

## Results

### Differences in relative expression of three alternative *BRD1* 5’ UTRs

**Fig 1:**
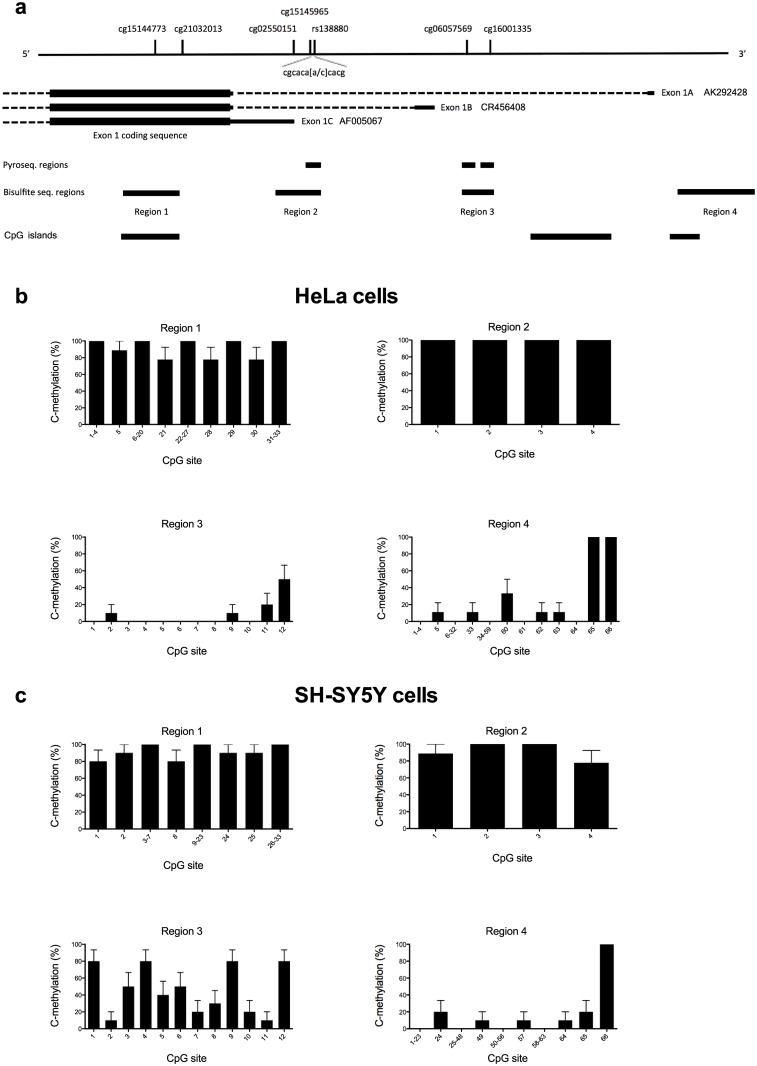
Structure of the *BRD1* promoter regions and methylation proportions in region 1–4 in cell lines. (a) Structure of the *BRD1* promoter regions. *BRD1* comprises 12 coding exons with the first coding exon 1 illustrated in the figure. *BRD1* was initially described to feature two splice variants, a long and a short resulting from alternative splicing of exon 7 and with transcription initiated from respectively, exon 1 with a 487 bp 5’ UTR (exon 1C) or from a non-coding exon located more than 3 kb upstream of the common exon 1 (exon 1A). We confirm the presence of a third non-coding exon located 1.4 kb upstream exon 1 (exon 1B). Additionaly, the alternative versions of exon 1 (exon 1C, 1B, and 1A) are supported by sequence evidence from different cell types including respectively, GenBank mRNA variant AF005067, CR456408, and AK292428 as well as EST sequences. On the figure the genomic localizations of SNP rs138880 and 6 CpG probes from the Illumina Infinium Human Methylation 450K Bead Array are illustrated: cg15144773, cg21032013, cg02550151, cg15145965, cg06057569, and cg16001335. For DNA methylation analysis, four regions (region 1–4) were analyzed by bisulfite sequencing. The number of CpG sites in each region is: region 1: 33, region 2: 4, region 3: 12, and region 4: 66. Region 2 and 3 was also examined by pyrosequencing. Pyrosequencing of region 2 included CpG sites 3 and 4 from the region 2 bisulfite sequencing assay, including cg15145965 (region 2, CpG site 3) and SNP rs138880. Pyrosequencing of region 3 covered the same region as examined by bisulfite sequencing but because of sequencing limitations it only included CpG sites 1–6 and 9–12. These CpG sites include cg06057569 (region 3, CpG site 6) and cg16001335 (region 3, CpG site 12). The genomic localizations of three CpG islands in the region are shown as well. (b) Methylation proportions as determined by bisulfite sequencing of region 1–4 in HeLa cells. Cells were cultured under standard conditions and DNA was isolated for bisulfite sequencing with primers listed in S3 Table. Data are presented as mean cytosine methylation proportions (C-methylation) at each CpG site +SEM (n = 9–10 clones for each region). For Region 1 and 4 CpG positions were merged when the methylation proportions were equal for adjacent sites. (c) Methylation proportions as determined by bisulfite sequencing of region 1–4 in SH-SY5Y cells using the same conditions as described for HeLa cells. Data are presented as mean cytosine methylation proportions (C-methylation) at each CpG site +SEM (n = 9–10 clones for each region).

In order to evaluate genomic regions in the *BRD1* locus for their potential importance in transcriptional regulation, we aimed to quantify the relative abundances of three different 5’ UTRs (exon 1C, 1B, and 1A) located upstream of exon 1 (Fig 1a) in RNA samples from HeLa (n = 3/group) and SH-SY5Y (n = 3/group) cells. The alternative versions of exon 1 (exon 1C, 1B, and 1A) are supported by evidence from respectively, GenBank mRNA variant AF005067, CR456408, and AK292428 as well as EST sequences. To quantify transcripts containing these different 5’ UTRs irrespective of which downstream exons were present primers were located internally in the exons (S2 Table). In HeLA cells each 5’ UTR comprised: exon 1A, 88.2%; exon 1B, 11%; and exon 1C, 0.8%. In SH-SY5Y cells each 5’ UTR comprised: exon 1A, 97.5%; exon 1B, 2.2%; and exon 1C, 0.3%. To further investigate whether transcripts containing exon 1A, 1B, and 1C are separate entities, we PCR amplified and DNA sequenced transcript regions starting at either exon 1A, 1B, or 1C and ending in exon 2 using oligo(dt) primed cDNA from HeLa and SH-SY5Y cells (S1 Fig). Amplification revealed in each case one major fragment of a size and DNA sequence in accordance with the following: exon 1A splicing to exon 1-exon 2; exon 1B splicing to exon 1-exon 2; and exon 1C splicing to exon 2, demonstrating that the 5’ UTRs are part of different transcripts. Similar results were obtained by amplifications ending in exon 4 and 6, respectively (S1 Fig), although longer fragments containing exon 1C were not efficiently amplified most likely due to its low expression levels. *BRD1* exists in a short and long transcript variant resulting from alternative splicing of exon 7 and it has been suggested that transcription from exon 1A results in the long variant whereas transcription from exon 1C results in the short variant [1]. Transcript predictions available e.g. in the Ensembl browser indicates the existence of up to 6 additional *BRD1* transcript variants including an exon 1C variant with a long version of exon 7. However, we note that the associated evidence for these 8 transcript variants does not clearly reveal whether certain 5’ UTRs are selectively associated with specific versions of exon 7. Additionally, this has neither been characterized in the cell lines investigated in the present study. In HeLa and SH-SY5Y cells, amplification from exon 1A and 1B ending in either exon 7 or 8 revealed in our experiments almost equal amplification of two fragments (S1 Fig). These fragments had sizes and DNA sequences (in those cases were they could be analyzed separately) comparing to respectively, long and short variants of exon 7. This suggests that differential splicing of exon 7 is independent of the actual 5’ UTR of the transcript.

### DNA methylation vary between *BRD1* alternative promoters and between cell lines

To investigate whether the differences in relative expression of the three 5’ UTRs are associated with differences in DNA methylation we performed bisulfite sequencing of four regions within and upstream of the *BRD1* gene (Fig 1a). For the 33 CpG sites investigated in region 1 (within the coding region of exon 1/exon 1C), the average CpG methylation proportion was 97.6% in HeLa cells (Fig 1b) and 97.3% in SH-SY5Y cells (Fig 1c). In region 2 (upstream of exon 1C), all four CpG sites were fully methylated in HeLa cells (Fig 1b) whereas in SH-SY5Y cells the average methylation proportion for the four CpG sites was 92.5% (Fig 1c). For region 3 (upstream of exon 1B), methylation proportions were low in HeLa cells at most CpG sites with an average of 7.5% (Fig 1b) whereas in SH-SY5Y cells, methylation was substantially higher with an average proportion of 45.8% (Fig 1c). For region 4 (upstream of exon 1A), methylation proportions were generally low in both cell lines with an average of 4.5% in HeLa cells (Fig 1b) and 2.6% in SH-SY5Y cells (Fig 1c). In this region, most methylation was attributed to sites that were specific to each cell line.

The co-occurrence of high DNA methylation proportions in region 2 and very low relative abundance of exon 1C containing transcripts (0.8% and 0.3%, respectively) suggests that DNA methylation could be a determining factor in transcriptional repression of this variant in HeLa and SH-SY5Y cells. DNA methylation in region 3 could play a role in the apparent differences in the transcription of the exon 1B containing variant between cell lines (11% and 2.2%, respectively), as higher DNA methylation proportions in SH-SY5Y cells corresponds with lower expression levels and *vice versa* in HeLa cells. In contrast, the high relative abundance of exon 1A containing transcripts in both cell lines (88.2% and 97.5%, respectively) suggests that the high methylation proportions of CpG site 65 and 66 in region 4 may not be important determinants for transcriptional regulation.

### Inhibition of DNA methylation is associated with increased *BRD1* transcription and reduced promoter methylation

**Fig 2:**
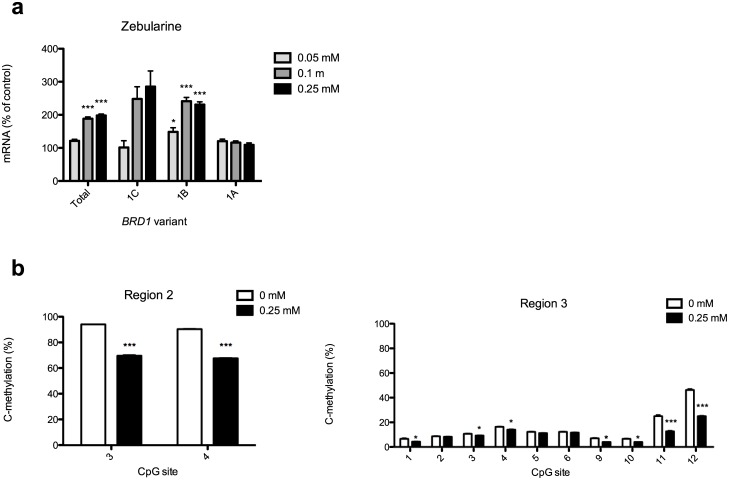
Expression of *BRD1* transcripts and methylation proportions in region 2 and 3 following Zebularine treatment in HeLa cells. (a) Expression of *BRD1* transcripts in HeLa cells following Zebularine treatment. The total *BRD1* expression and expression of transcript variants containing exon 1C, 1B, and 1A were measured in RNA extracted from HeLa cells following exposure to 0, 0.05, 0.1, or 0.25 mM Zebularine for 72 hours. *PGK1* and *TBP* were found to be the most stably expressed reference genes and were used for normalization. Data are presented as mean percentages of the mean value of the control group (no Zebularine treatment) +SEM (n = 3/group). *p<0.05, ***p<0.001, one-way ANOVA with Dunnett's post-hoc tests. (b) Methylation proportions as determined by pyrosequencing of region 2 and 3 following Zebularine treatment in HeLa cells. Methylation proportions were measured by pyrosequencing of region 2 and 3 in DNA extracted from HeLa cells following exposure to 0 or 0.25 mM Zebularine for 72 hours. Data are presented as mean cytosine methylation proportions (C-methylation) at each CpG site +SEM (n = 3/group). *p<0.05, ***p<0.001, unpaired t-test.

To confirm that DNA methylation is important for transcriptional regulation of *BRD1*, HeLa and SH-SY5Y cells were treated with the DNA methyltransferase (DNMT) inhibitor Zebularine for 72 hours and the relative expression of different *BRD1* exons were measured using qPCR. In HeLa cells, the total *BRD1* expression (as measured by quantification of transcripts containing the exon 11–12 boundary) increased 100% above the levels measured in untreated cells (*p*<0.001) at Zebularine doses of 0.25 mM (Fig 2a). This was accompanied by a 131% increase in expression of exon 1B containing transcripts (*p*<0.001). Although the expression of exon 1C containing transcripts under the same conditions were 185% above the levels measured in untreated cells the difference was not statistically significant likely due to high variance arising from its low expression levels (Fig 2a). In SH-SY5Y cells there was no significant effect of Zebularine on the expression of any of the *BRD1* transcripts investigated (S2 Fig).

To investigate whether Zebularine actually affects DNA methylation in the *BRD1* locus, we measured changes in DNA methylation upon treatment of HeLa cells with 0.25 mM Zebularine. Because of the increase in expression of exon 1C and 1B containing transcripts upon Zebularine treatment (Fig 2a), we focused on the genomic regions immediately upstream of these exons, that is region 2 and 3, respectively (Fig 1a). Pyrosequencing assays revealed methylation patterns in untreated cells (Fig 2b) that were consistent with those observed by bisulfite sequencing (Fig 1b). The analysis further revealed that Zebularine had profound effects on DNA methylation in both regions. For region 2, Zebularine treatment resulted in a significant reduction in methylation proportions from 94.0% to 69.7% (*p*<0.001) at CpG site 3 and a reduction from 90.3% to 67.7% at site 4 (*p*<0.001) (Fig 2b). For Region 3, a significant reduction from 6.7% to 4.3% was observed at CpG site 1 (*p*<0.05), from 10.7% to 9.3% at site 3 (*p*<0.05), from 16.3% to 14.0% at site 4 (*p*<0.05), from 7.0% to 4.0% at site 9 (*p*<0.05), from 6.7% to 4.0% at site 10 (*p*<0.05), from 25.0% to 12.7% at site 11 (*p*<0.001), and from 46.3% to 25.0% at site 12 (*p*<0.001) (Fig 2b). Thus, although some of the differences in region 3 were very small, the increase in expression of transcripts containing exon 1C and 1B in general corresponds with reduced methylation proportions in their upstream genomic regions.

### Association between the rs138880 risk allele and increased DNA methylation in adipose tissue

Because of the genomic location of the schizophrenia rs138880 risk variant immediately upstream of exon 1C (Fig 1a) and the observation that it is associated with reduce *BRD1* expression [14], we decided to investigate if it is also associated with increased methylation in its vicinity in a public available dataset including Ilumina 450K adipose methylome data from 648 twins (the Multiple Tissue Human Expression Resource (MuTHER)) [27]. MetQTL analysis encompassing a distance up to 10 kb surrounding the SNP (+/- 5 kb) using the Genevar (GENe Expression VARiation) platform [25] revealed that the risk allele was significantly associated with increased methylation at three probe sites (*p*-value threshold = 0.001): cg02550151, cg15145965, and cg06057569 (Fig 1a). As cg02550151 and cg15145965 are located in genomic region 2 and cg06057569 in region 3, it is possible that increased methylation of these sites represses transcription of *BRD1* in risk allele carriers.

### The rs138880 risk allele is associated with moderate regional *BRD1* promoter hypermethylation in blood

**Fig 3:**
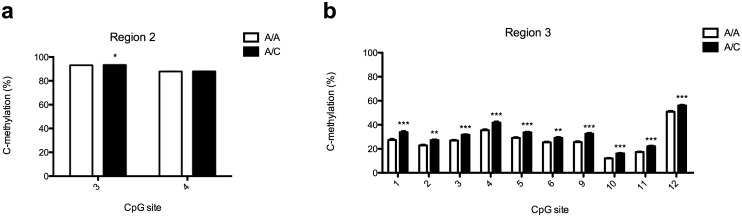
Methylation proportions in region 2 and 3 in blood from healthy individuals. Methylation proportions as determined by pyrosequencing of region 2 and 3 in blood from healthy individuals. Methylation proportions were measured by pyrosequencing of region 2 and 3 in DNA extracted from blood obtained from healthy females being either homozygous (A/A) or heterozygous (A/C) for SNP rs138880. The assay designed for region 2 allowed confirmation of the rs138880 genotype. Data are presented as mean cytosine methylation proportions (C-methylation) at each CpG site +SEM (n = 24/group). *p<0.05, **p<0.01, ***p<0.001, unpaired t-test.

To assess whether DNA methylation may also be correlated to the rs138880 genotype in other tissues, we measured methylation in DNA extracted from blood obtained from 48 healthy females; 24 homozygous (A/A) and 24 heterozygous (A/C) that had previously been genotyped [9]. Since the sex of the subjects was recorded in the biobank, we were able to obtain a homogenous collection of samples in relation to this parameter, however only females were analysed since they were far more abundant in the biobank than males. Pyrosequencing was used to measure DNA methylation proportions at several sites in region 2 and 3 (Fig 1a), including cg15145965 (region 2, CpG site 3) and cg06057569 (region 3, CpG site 6). In addition, the pyrosequencing assay for region 2 was designed to allow confirmation of the rs138880 genotype (S3 Fig). In region 2, we observed that both investigated CpG sites were highly methylated in both A/A and A/C individuals with levels above 93% for CpG site 3 and above 88% for CpG site 4 (Fig 3). Additionally, for CpG site 3 we observed a very modest though significant difference in DNA methylation with a methylation proportion of 93.08% in A/A individuals compared to 93.38% in A/C individuals (*p*<0.05). In region 3, we observed a larger variation in methylation proportions with levels ranging from 12% to 51% at the investigated CpG sites in A/A individuals (Fig 3). Importantly, we found that at all the 10 investigated CpG sites, methylation proportions were significantly higher (between 3.6% and 6.9%) in A/C compared to A/A individuals (Fig 3). Thus, it seems that risk allele carriers have slightly increased DNA methylation in the *BRD1* locus also in blood, a finding that agrees well with the approximately 3% reduction in *BRD1* mRNA expression found in B lymphoblastoid cell lines derived from individuals of the same rs138880 genotype [14].

### Risk allele hypermethylated regions undergo epigenetic changes during brain development

**Fig 4:**
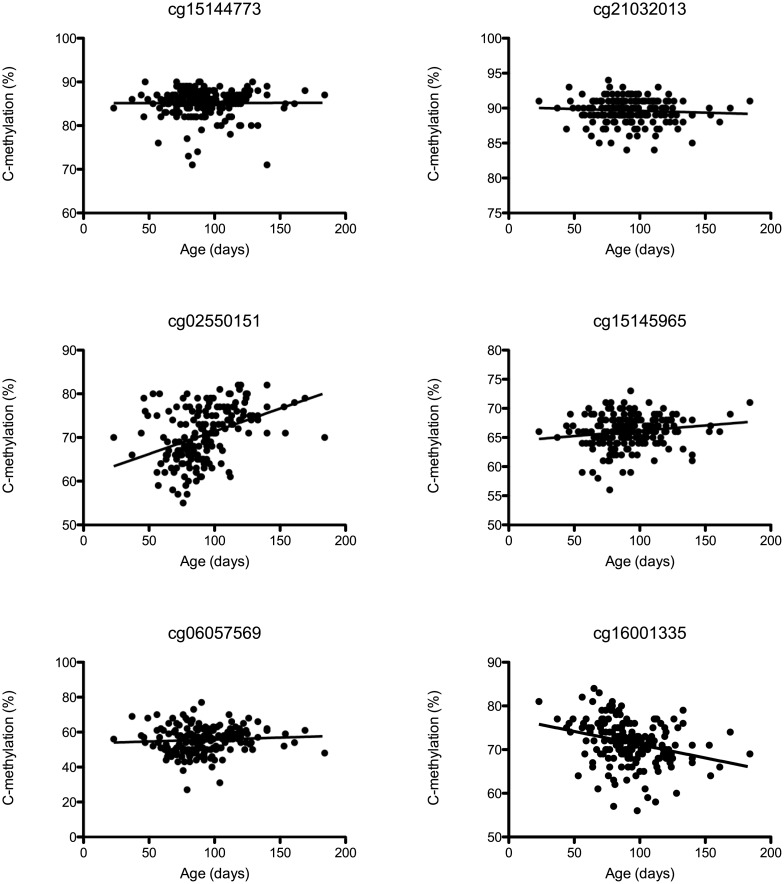
Methylation proportion changes in the *BRD1* locus during fetal neocortex development. Methylation proportions in the *BRD1* locus during fetal neocortex development. A public available dataset was utilized to correlate DNA methylation proportions with fetal age (n = 179, range = 23–184 days post-conception). DNA methylation data was available for probes from the Illumina Infinium Human Methylation 450K Bead Array. Probes located near the promoter region of *BRD1* were assessed for correlation between DNA methylation proportions and fetal age. All probes that revealed age related changes in methylation proportions and some that did not are illustrated. Statistical analysis was performed with Pearson’s correlation test.

It has previously been suggested that *BRD1* plays an important role in neurodevelopment [3,8] and reduced levels of *BRD1* could be a risk factor in schizophrenia with a pathogenic mechanism in line with the neurodevelopmental hypothesis of the disorder [28]. To examine if genomic regions being hypermethylated in rs138880 risk allele carriers undergo methylation changes during fetal brain development, we assessed the correlation between DNA methylation proportions near the promoter region of *BRD1* and fetal age in an online dataset with Ilumina 450K brain methylome data from 179 fetuses (range = 23–184 days post-conception) [29]. Two probes, cg15144773 and cg21032013 located in exon 1/exon 1C (Fig 1a) revealed no correlation (Fig 4). Two probes located immediately upstream of exon 1C (Fig 1a) revealed significant increases in DNA methylation proportions with increasing fetal age, cg02550151 (*p*<0.0001, r = 0.41) and cg15145965 (*p*<0.05, r = 0.16) (Fig 4). Among probes located upstream of exon 1B (Fig 1a), one revealed no correlation (cg06057569) whereas the other, cg16001335 revealed significant decreases in methylation with increasing fetal age (*p*<0.0001, r = 0.30) (Fig 4). To further explore whether these risk allele hypermethylated regions undergo epigenetic changes also when comparing fetal and adult frontal cortex, we extracted summary statiscs data for the same probes (S4 Table) from a recent DNA methylation study including n = 35 fetal and n = 300 postnatal samples [18]. Interestingly, we find significant (although small to moderate) differences in DNA methylation proportions for 5 out of the 6 probes (cg15144773, cg02550151, cg15145965, cg06057569, and cg16001335, *p*<0.001 for all). The largest differences in methylation proportions (and the most significant) are observed for the cg06057569 and cg16001335 probes in region 3 (with the fetal brains having higher methylation proportion than postnatal brains), hereafter the differences in methylation observed for the cg02550151 and cg15145965 probes in region 2 (also with the fetal brains having higher methylation proportion than postnatal brains), whereas the methylation differences observed for the cg15144773 and cg21032013 probes in region 1 are, respectively, only very slight (with the fetal brains having lower methylation proportions than the postnatal brains) or not significant. Of further note, there seems to be a significant and high correlation between DNA methylation differences in both region 2 and region 3 and differences in *BRD1* expression assessed in the same samples (*p*<0.001 for all), especially for DNA methylation differences observed with the cg16001335 probe in region 3, *p* = 9.86E-26. Unfortunately, we were not able to perform cis-MetQTL analysis of the rs138880 SNP in this dataset since it was not genotyped.

### Commonly used mood stabilizers differently affect *BRD1* transcription

**Fig 5:**
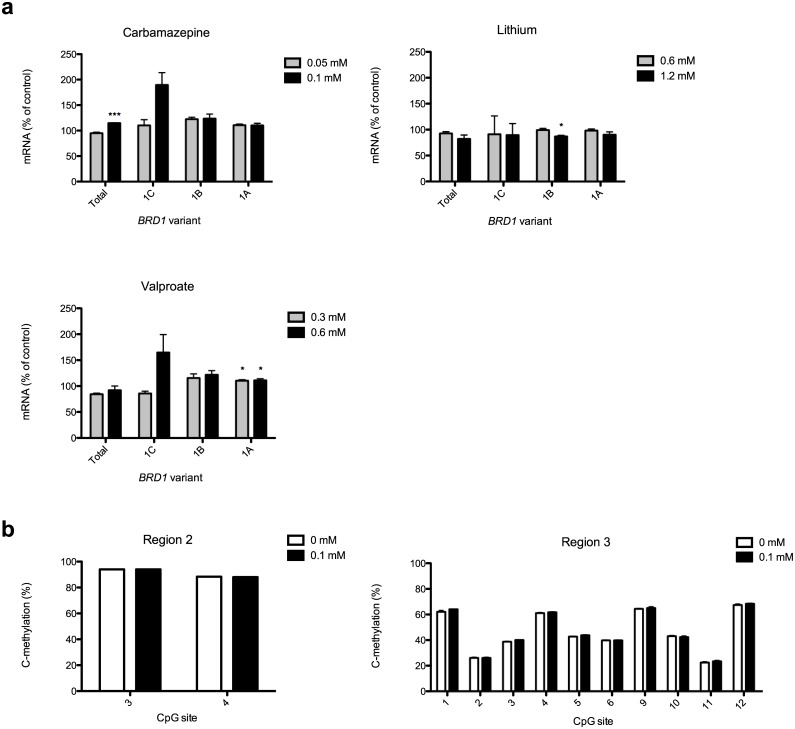
Expression of *BRD1* transcripts and methylation proportions in region 2 and 3 in SH-SY5Y cells following drug treatment. (a) Expression of *BRD1* transcripts in SH-SY5Y cells following Lithium, Valproate, and Carbamazepine treatment. The total *BRD1* expression and expression of transcript variants containing exon 1C, 1B, and 1A were measured in RNA extracted from control cells or following exposure of the cells to the lowest and highest therapeutic dosage of the drugs for 72 hours in SH-SY5Y cells. Data are presented as mean percentages of the mean value of the control group (no drug treatment) +SEM (n = 3/group). ***p<0.001, one-way ANOVA with Dunnett's post-hoc tests. (b) Methylation proportions as determined by pyrosequencing of region 2 and 3 following Carbamazepine treatment in SH-SY5Y cells. Methylation proportions were measured by pyrosequencing of region 2 and 3 in DNA extracted from SH-SY5Y cells following exposure to 0 or 0.1 mM Carbamazepine for 72 hours. Data are presented as mean cytosine methylation proportions (C-methylation) at each CpG site +SEM (n = 3/group). Group means were compared by unpaired t-test.

It has been suggested that upregulation of *BRD1* is involved in stress resilience and response to antidepressants [5,6] and reduced risk for schizophrenia [14]. Thus, we decided to investigate if therapeutic doses of three commonly used mood stabilizers that have profound effect on DNA methylation levels [19] affect expression of *BRD1* in SH-SY5Y cells. We find that SH-SY5Y cells due to their origin (neuroblastoma) and neuronal characteristics represent a better model than HeLA cells to investigate whether brain directed drugs (mood stabilizers) would result in increased expression of *BRD1* and associated changes in methylation. Generally, the mood stabilizers caused diverse effects on the expression of *BRD1* as measured using qPCR (Fig 5a). For Lithium, the highest dosage (1.2 mM) caused a 13% decrease in the expression of exon 1B containing transcripts (*p*<0.05). Valproate, both 0.3 mM and 0.6 mM, caused an approx. 10% increase in the expression of exon 1A containing transcripts (*p*<0.05). Carbamazepine in a dosage of 0.1 mM caused a 15% increase in the expression of total *BRD1* (*p*<0.001). In the latter case, the changes in the expression of the alternative exon 1 variants were not significant albeit tendencies for increased expression of 90%, 24%, and 10% for exon 1C, 1B, and 1A, respectively, were observed (Fig 5a). To dissect mechanisms of action of the mood stabilizer causing the most profound transcriptional upregulation of *BRD1*, we measured methylation proportions in DNA extracted from cells treated with 0.1 mM Carbamazepine for 72 hours with the pyrosequencing assays designed for region 2 and 3. The pyrosequencing assays revealed methylation patterns (Fig 5b) consistent with those observed by bisulfite sequencing in untreated cells (Fig 1c), however Carbamazepine treatment in SH-SY5Y cells had no effect on DNA methylation proportions at any of the investigated CpG sites (Fig 5b). Thus, these results indicate that Carbamazepine most likely affects *BRD1* transcription in SH-SY5Y cells by another mechanism.

## Discussion

We present evidence that *BRD1* transcription is initiated from three alternative promoters that show large differences in activity and might be regulated by DNA methylation. We also find that the schizophrenia-associated C allele of rs138880 not only correlates with slightly reduced *BRD1* expression but also with moderately increased DNA methylation in *BRD1* promoter regions in both adipose tissue and blood. Interestingly, we find that these risk allele hypermethylated regions undergo changes in methylation during brain development and that these changes correlate well with changes in *BRD1* expression. Finally, we demonstrate that commonly used mood stabilizers affect the expression of *BRD1* in SH-SY5Y cells, however most likely by mechanisms other than DNA methylation changes in the promoter regions studied.

For the genomic regions upstream of exon 1A and exon 1B, data from The ENCODE Project: ENCyclopedia Of DNA Elements [30] show binding of RNA Polymerase II, large clusters of transcription factors as well as acetylation of lysine 27 of the H3 histone protein, all of which support our evidence that these regions participate in transcriptional regulation of *BRD1* in cell lines. For the exon 1C containing transcript variant we find very low expression in both cell lines investigated, however still the genomic region located upstream do show promoter activity in murine neuroblastoma cells [14]. Our finding that exon 1A and 1B are part of both long and short exon 7 transcript variants encoding protein isoforms with clearly distinct properties [1,4] indicate that the 5’ UTRs are more likely to be important for developmental and/or tissue specific expression than for alternative splicing [31].

When genes have several promoters, DNA methylation may serve as a mechanism for controlling alternative promoter usage [32]. In HeLa cells treated with Zebularine we observed that methylation covaried negatively with expression of exon 1B containing transcripts whereas it covaried only somewhat or not at all with the expression of exon 1A containing transcripts. This is likely because the sites in region 3 with highest methylation proportions are located approximately 400 bp upstream of exon 1B (CpG site 11 and 12) whereas in region 4, the sites with highest methylation proportions are located approximately 700 bp upstream of exon 1A (CpG sites 65 and 66). Thus, CpG site 65 and 66 in region 4 may rather be important for creating a boundary and stabilizing the nucleosomes at the interface before the potentially nucleosome depleted region [33,34]. Indeed most cell lines examined in The ENCODE Project are highly methylated at CpG site 66. In HeLa cells, Zebularine treatment increased expression of exon 1C and decreased methylation of CpG site 3 and 4 in region 2. This emphasizes that DNA methylation at these sites are most likely repressing transcription but several other CpG sites located in the region could be equally important. In SH-SY5Y cells we observe no effect of Zebularine on *BRD1* expression. Zebularine blocks DNMT activity and this prevents maintenance methylation during DNA replication. Therefore, the most likely explanation for the lack of effect in SH-SY5Y cells is that the growth rate is substantially higher in HeLa than in SH-SY5Y cells resulting in a higher loss of methylation upon treatment.

Much effort put into identification of risk factors for schizophrenia has revealed SNPs significantly associated with the disorder however studies are needed to reveal their mechanistic contribution to the pathogenesis. We have recently demonstrated that the risk allele of rs138880 located in one of the *BRD1* promoter regions very close to the transcription start site causes a small (approximately 3%) but significant decrease in the expression of *BRD1* in B lymphoblastoid cell lines [14]. In addition, the risk allele has lower transcriptional drive in a neuronal cell line (approximately 23% compared to the A allele) [14]. Here we find that the risk allele of rs138880 (the C allele) is associated with a moderate increased methylation in region 3. This genomic region is located in the potential promoter region of the exon 1B containing transcript variant. Based on our data, DNA methylation in this specific genomic region may affect the expression of the exon 1B containing transcript variant. With this transcript being an apparent minor variant in cell lines, it may explain why the effect of the risk allele on *BRD1* expression is small in B lymphoblastoid cell lines. This said, the rs138880 SNP is also significantly associated with expression of other genes e.g. in cerebellum it seems to associate with eventual expression of a lincRNA (RP3-522J7.6) encoded by a locus located approximately 13 kb further upstream of the *BRD1* promoter region. This association could contribute to further effects on epigenetic regulation, however due to the design of our experiments this can not be further enlightened in the present study.

The risk allele of rs138880 is associated with moderately increased methylation at several CpG sites in region 3 and the reason for this is currently unknown. However, evidence suggests that DNA methylation could be a secondary event following decreased promoter activity [35]. Active promoter regions are nucleosome-depleted [36] and it has been found that DNMT binding requires nucleosomes and that binding cannot occur if the nucleosomes are marked with the activating histone marks H3K4me2 or H3K4me3 [37]. Thus in the case of *BRD1*, it might be that methylation is a secondary consequence of decreased binding of a transcriptional activator or increased binding of a transcriptional repressor to the risk allele. This is supported by other studies, for example the finding that a SNP in the promoter region of the *MLH1* gene caused a decreased transcriptional drive and the less active allele was more likely to become methylated in somatic cells [38]. Previous comparisons of predicted transcription factor binding at the genomic region surrounding rs138880 with the MatInspector software [39] suggested that the transcriptional repressor HES1 could bind specifically to the risk allele [8]. In addition, the same software predicts that BPFT and RBP2 could bind specifically to the A-allele wheras the HaploReg tool predicts differential binding of BRCA1, FAC1 and Egr-1 to the two variant alleles [40]. Thus, it might be that differences in transcription factor binding decrease transcriptional activity of the risk allele and lead to increased methylation however direct experimental data to support this is lacking.

In the developing human fetal brain, *BRD1* is expressed more at early embryonic stages than in the later stages [4]. Interestingly, the two *BRD1* promoter regions exhibiting moderately increased DNA methylation in adipose tissue and blood from carriers of the rs138880 risk allele co-localize with CpG sites undergoing DNA methylation changes during fetal brain development and being differently methylated and associated with changes in *BRD1* expression in fetal and adult frontal cortex. Based on the inverse methylation changes—with increasing methylation at the promoter region upstream of exon 1C and decreasing methylation at the promoter region upstream of exon 1B it could be speculated that expression of the exon 1C containing transcript variant is higher in early fetal brain and then gradually decreases whereas for exon 1B it is the opposite. It is currently unknown whether the rs138880 risk allele influences DNA methylation in fetal brains but importantly, if the same phenomenon (that is hypermethylation in region 2 and 3 of the risk allele) occur in fetal brains it would suggest that expression of the exon 1C containing transcript variant is repressed during early fetal development in risk allele carriers and exon 1B expression is repressed later in fetal development and persisting into adulthood. Such dysregulation of *BRD1* could adversely influence expression of several genes being important for normal brain development since *BRD1* has been found to bind to the promoter regions and potentially regulate the transcription of a number of schizophrenia risk genes [4].

In conclusion, our studies have provided new knowledge regarding the transcriptional regulation of the *BRD1* gene. This is particularly interesting in relation to neurodevelopment and mental disorders since *BRD1* has been implicated in both. Not only is it possible that dysregulation of *BRD1* leads to neurodevelopmental disturbances but it may be equally important in the adult brain as increased DNA methylation may prevent stress induced *BRD1* upregulation and stress adaptions. Future studies may reveal whether modulation of *BRD1* expression is relevant as a therapeutic target.

## Supporting Information

S1 Fig PCR amplification of transcript regions. (a) PCR amplification of transcript regions starting at exon 1A. Left gel: HeLa cells, right gel: SH-SY5Y cells. Lane 1: 1 kb DNA ladder (Thermo Scientific), lane 2: exon 1A F + exon 2 R, lane 3: exon 1A F + exon 4 R, lane 4: exon 1A F + exon 6 R, lane 4: exon 1A F + exon 7 R, lane 5: exon 1A F + exon 8 R. (b) PCR amplification of transcript regions starting at exon 1B. Left gel: HeLa cells, right gel: SH-SY5Y cells. Lane 1: 1 kb DNA ladder, lane 2: exon 1B F + exon 2 R, lane 3: exon 1B F + exon 4 R, lane 4: exon 1B F + exon 6 R, lane 4: exon 1B F + exon 7 R, lane 5: exon 1B F + exon 8 R. (c) PCR amplification of transcript regions starting at exon 1C. Left gel: HeLa cells, right gel: SH-SY5Y cells. Lane 1: 1 kb DNA ladder, lane 2: exon 1C F + exon 2 R, lane 3: exon 1C F + exon 4 R, lane 4: exon 1C F + exon 6 R, lane 4: exon 1C F + exon 7 R, lane 5: exon 1C F + exon 8 R.
(DOCX)

S2 Fig Expression of *BRD1* transcript variants in SH-SY5Y cells following Zebularine treatment. The total *BRD1* expression and expression of transcript variants containing exon 1C, 1B, and 1A were measured in RNA extracted from SH-SY5Y cells following exposure to 0, 0.05, 0.1, or 0.25 mM Zebularine for 72 hours. *POLR2A* and *TBP* were found to be the most stably expressed reference genes and were used for normalization. Data are presented as mean percentages of the mean value of the control group (no Zebularine treatment) +SEM (n = 3/group). Values were compared by one-way ANOVA with Dunnett’s post-hoc test.
(DOCX)

S3 Fig Region 2 pyrosequencing assay example. A representative pyrogram from a region 2 assay run on a homozygous (A/A) individual. CpG methylation proportions were measured at two sites, CpG sites 3 and 4 marked with blue columns at dispensation 9–10 and 20–21, respectively. In this example methylation proportions are 93% at site 3 and 88% at site 4. At dispensation 16 and 17 it is clear that this individual is homozygous (A/A). In case an individual is heterozygous (A/C), cytosine would have been converted to thymine thereby resulting in similarly sized A and T peaks. Oppositely, a homozygous (C/C) individual would display a large T peak at dispensation 17, however no such individuals were present in the study population. The red bar at dispensation 5 is a bisulfite conversion control, clearly demonstrating that unmethylated cytosine has been fully converted.
(DOCX)

S1 Supporting Information Supplementary materials and methods. Detailed description of the materials and methods used for transcript variant analysis.
(DOCX)

S1 Table Primer sequences, expected amplicon sizes, and PCR extension times for transcript variant analysis. (DOCX)

S2 Table Primer sequences and expected amplicon sizes for real-time qPCR. (DOCX)

S3 Table Primer sequences, expected amplicon sizes, and PCR annealing temperatures for bisulfite sequencing and pyrosequencing. (DOCX)

S4 Table Summary statistics for 6 selected DNA methylation differences between fetal and postnatal human cortex and their correlation to *BRD1* expression. (DOCX)
