# Supplementary material for: DNA Methylation Analysis of BRD1 Promoter Regions and the Schizophrenia rs138880 Risk Allele
Source: PLoS One. 2017 Jan 17;12(1):e0170121. doi: 10.1371/journal.pone.0170121 (PMC5240986; doi:10.1371/journal.pone.0170121)
Supplement: S1 Fig — (a) PCR amplification of transcript regions starting at exon 1A. Left gel: HeLa cells, right gel: SH-SY5Y cells. Lane 1: 1 kb DNA ladder (Thermo Scientific), lane 2: exon 1A F + exon 2 R, lane 3: exon 1A F + exon 4 R, lane 4: exon 1A F + exon 6 R, lane 4: exon 1A F + exon 7 R, lane 5: exon 1A F + exon 8 R. (b) PCR amplification of transcript regions starting at exon 1B. Left gel: HeLa cells, right gel: SH-SY5Y cells. Lane 1: 1 kb DNA ladder, lane 2: exon 1B F + exon 2 R, lane 3: exon 1B F + exon 4 R, lane 4: exon 1B F + exon 6 R, lane 4: exon 1B F + exon 7 R, lane 5: exon 1B F + exon 8 R. (c) PCR amplification of transcript regions starting at exon 1C. Left gel: HeLa cells, right gel: SH-SY5Y cells. Lane 1: 1 kb DNA ladder, lane 2: exon 1C F + exon 2 R, lane 3: exon 1C F + exon 4 R, lane 4: exon 1C F + exon 6 R, lane 4: exon 1C F + exon 7 R, lane 5: exon 1C F + exon 8 R. (DOCX) [file pone.0170121.s001.docx]

**a b c**


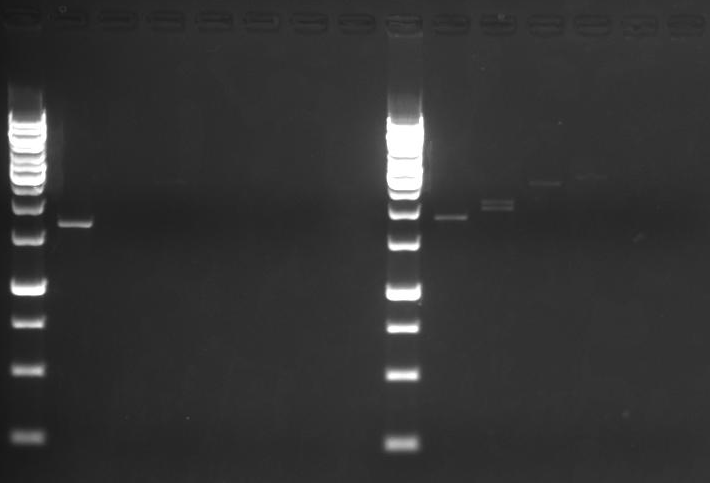

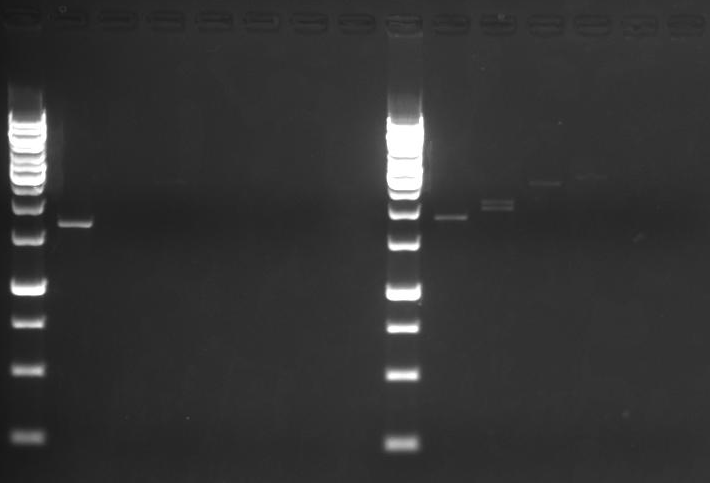
**
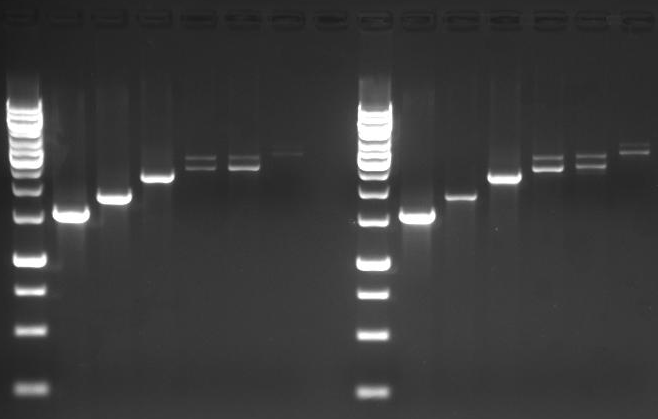

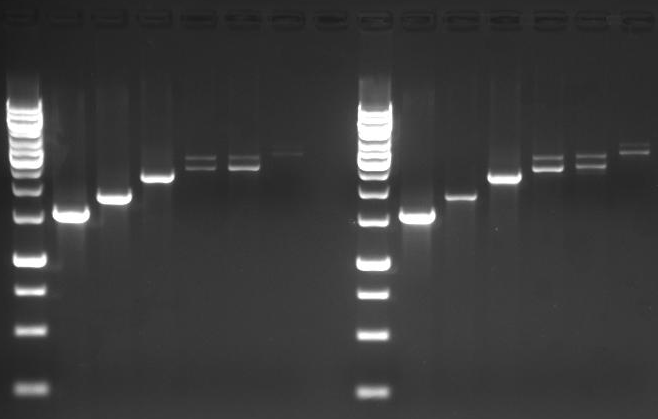

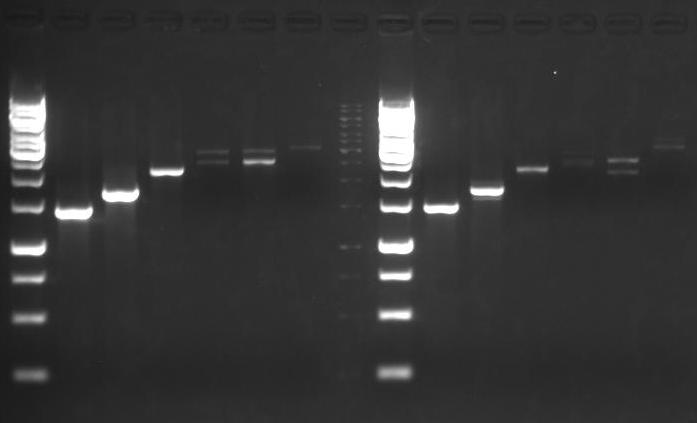

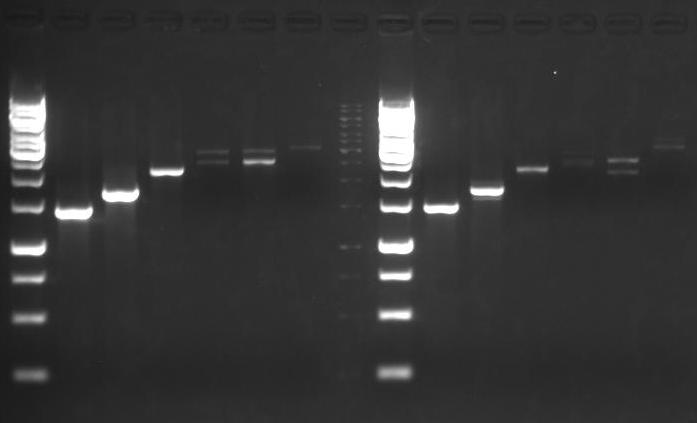
**

**S1 Fig. PCR amplification of transcript regions.** (a) PCR amplification of transcript regions starting at exon 1A. Left gel: HeLa cells, right gel: SH-SY5Y cells. Lane 1: 1 kb DNA ladder (Thermo Scientific), lane 2: exon 1A F + exon 2 R, lane 3: exon 1A F + exon 4 R, lane 4: exon 1A F + exon 6 R, lane 4: exon 1A F + exon 7 R, lane 5: exon 1A F + exon 8 R. (b) PCR amplification of transcript regions starting at exon 1B. Left gel: HeLa cells, right gel: SH-SY5Y cells. Lane 1: 1 kb DNA ladder, lane 2: exon 1B F + exon 2 R, lane 3: exon 1B F + exon 4 R, lane 4: exon 1B F + exon 6 R, lane 4: exon 1B F + exon 7 R, lane 5: exon 1B F + exon 8 R. (c) PCR amplification of transcript regions starting at exon 1C. Left gel: HeLa cells, right gel: SH-SY5Y cells. Lane 1: 1 kb DNA ladder, lane 2: exon 1C F + exon 2 R, lane 3: exon 1C F + exon 4 R, lane 4: exon 1C F + exon 6 R, lane 4: exon 1C F + exon 7 R, lane 5: exon 1C F + exon 8 R.
