# Supplementary material for: DNA Methylation Analysis of BRD1 Promoter Regions and the Schizophrenia rs138880 Risk Allele
Source: PLoS One. 2017 Jan 17;12(1):e0170121. doi: 10.1371/journal.pone.0170121 (PMC5240986; doi:10.1371/journal.pone.0170121)
Supplement: S2 Fig — The total BRD1 expression and expression of transcript variants containing exon 1C, 1B, and 1A were measured in RNA extracted from SH-SY5Y cells following exposure to 0, 0.05, 0.1, or 0.25 mM Zebularine for 72 hours. POLR2A and TBP were found to be the most stably expressed reference genes and were used for normalization. Data are presented as mean percentages of the mean value of the control group (no Zebularine treatment) +SEM (n = 3/group). Values were compared by one-way ANOVA with Dunnett’s post-hoc test. (DOCX) [file pone.0170121.s002.docx]

**S2 Fig. Expression of *BRD1* transcript variants in SH-SY5Y cells following zebularine treatment.** The total *BRD1* expression and expression of transcript variants containing exon 1C, 1B, and 1A were measured in RNA extracted from SH-SY5Y cells following exposure to 0, 0.05, 0.1, or 0.25 mM zebularine for 72 hours. *POLR2A* and *TBP* were found to be the most stably expressed reference genes and were used for normalization. Data are presented as mean percentages of the mean value of the control group (no Zebularine treatment) +SEM (n = 3/group). Values were compared by one-way ANOVA with Dunnett’s post-hoc test.
