# Supplementary material for: DNA Methylation Analysis of BRD1 Promoter Regions and the Schizophrenia rs138880 Risk Allele
Source: PLoS One. 2017 Jan 17;12(1):e0170121. doi: 10.1371/journal.pone.0170121 (PMC5240986; doi:10.1371/journal.pone.0170121)
Supplement: S3 Fig — A representative pyrogram from a region 2 assay run on a homozygous (A/A) individual. CpG methylation proportions were measured at two sites, CpG sites 3 and 4 marked with blue columns at dispensation 9–10 and 20–21, respectively. In this example methylation proportions are 93% at site 3 and 88% at site 4. At dispensation 16 and 17 it is clear that this individual is homozygous (A/A). In case an individual is heterozygous (A/C), cytosine would have been converted to thymine thereby resulting in similarly sized A and T peaks. Oppositely, a homozygous (C/C) individual would display a large T peak at dispensation 17, however no such individuals were present in the study population. The red bar at dispensation 5 is a bisulfite conversion control, clearly demonstrating that unmethylated cytosine has been fully converted. (DOCX) [file pone.0170121.s003.docx]

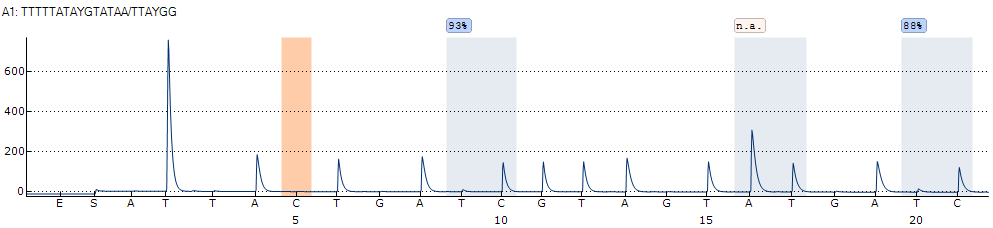


**S3 Fig. Region 2 pyrosequencing assay example.** A representative pyrogram from a region 2 assay run on a homozygous (A/A) individual. CpG methylation proportions were measured at two sites, CpG sites 3 and 4 marked with blue columns at dispensation 9-10 and 20-21, respectively. In this example methylation proportions are 93% at site 3 and 88% at site 4. At dispensation 16 and 17 it is clear that this individual is homozygous (A/A). In case an individual is heterozygous (A/C), cytosine would have been converted to thymine thereby resulting in similarly sized A and T peaks. Oppositely, a homozygous (C/C) individual would display a large T peak at dispensation 17, however no such individuals were present in the study population. The red bar at dispensation 5 is a bisulfite conversion control, clearly demonstrating that unmethylated cytosine has been fully converted.
