# Supplementary material for: DNA Methylation Analysis of BRD1 Promoter Regions and the Schizophrenia rs138880 Risk Allele
Source: PLoS One. 2017 Jan 17;12(1):e0170121. doi: 10.1371/journal.pone.0170121 (PMC5240986; doi:10.1371/journal.pone.0170121)
Supplement: S1 Supporting Information — Detailed description of the materials and methods used for transcript variant analysis. (DOCX) [file pone.0170121.s004.docx]

**S1 Supporting Information. Supplementary materials and methods.** Detailed description of the materials and methods used for transcript variant analysis.

**cDNA synthesis**

1 μg of RNA was DNase digested with DNase I (Thermo Scientific, Germany), according to manufacturer's instructions. 1 μg of RNA was reverse transcribed with a Maxima H Minus First Strand cDNA Synthesis Kit (Thermo Scientific), using the protocol for RT-PCR with oligo (dT)_18_ primers. No reverse transcriptase control reactions were prepared identically but without addition of Maxima H Minus Enzyme Mix. The reactions were incubated at 50°C for 30 min, 65°C for 30 min, and 85 °C for 5 min. cDNA and no reverse transcriptase reactions were diluted 1:40 with H_2_O and used directly as PCR templates.

**PCR conditions**

*BRD1* transcripts were amplified using HotStarTaq DNA Polymerase (Qiagen, Hilden, Germany) in 20 μL reactions with minor deviations to the standard protocol: 10 μL template, 0.5 μM of each primer, 300 μM of each dNTP and 1.5 mM MgCl_2_.The cycling conditions were: 95°C for 15 min, 40x (94°C 30 sec, 65°C x min, 72**°**C 3 or 5 min), 72°C 10 min. Primer sequences, expected amplicon sizes and PCR extension times are listed in S1 Table.

**Direct DNA sequencing**

Selected fragments were cut from the agarose gel and isolated with a QIAquick Gel Extraction Kit (Qiagen). Purified PCR products were sequenced with the reverse primer used for its initial amplification (GATC Biotech, Constance, Germany).
