# Supplementary material for: DNA Methylation Analysis of BRD1 Promoter Regions and the Schizophrenia rs138880 Risk Allele
Source: PLoS One. 2017 Jan 17;12(1):e0170121. doi: 10.1371/journal.pone.0170121 (PMC5240986; doi:10.1371/journal.pone.0170121)
Supplement: S1 Table — (DOCX) [file pone.0170121.s005.docx]

**S1 Table.** **Primer sequences, expected amplicon sizes, and PCR extension times for transcript variant analysis.**

| Primer name | Primer sequence (5’>3’) | Amplicon size (bp) | Extension  time (min) |
| --- | --- | --- | --- |
| Exon 1A  Exon 2 | Forward: CGGCGCCCCGAAGGTAATC  Reverse: GCCGCTTGAGCAGCCAGTAG | 1480 | 3 |
| Exon 1A  Exon 4 | Forward: CGGCGCCCCGAAGGTAATC  Reverse: GCGGGGTCCTTGTCTTGCAG | 1778 | 3 |
| Exon 1A  Exon 6 | Forward: CGGCGCCCCGAAGGTAATC  Reverse: TTTGAGCAGCTTTGCCCGCTT | 2262 | 5 |
| Exon 1A  Exon 7 | Forward: CGGCGCCCCGAAGGTAATC  Reverse: TCCAGGCGCTTTCCTGGGGA | 2486/2879 | 5 |
| Exon 1A  Exon 8 | Forward: CGGCGCCCCGAAGGTAATC  Reverse: CTCCTGCTCGCTCCTCGCA | 2532/2925 | 5 |
| Exon 1B  Exon 2 | Forward: ATACCCAGTGTCTGGAGGGTCTG  Reverse: GCCGCTTGAGCAGCCAGTAG | 1589 | 3 |
| Exon 1B  Exon 4 | Forward: ATACCCAGTGTCTGGAGGGTCTG  Reverse: GCGGGGTCCTTGTCTTGCAG | 1887 | 3 |
| Exon 1B  Exon 6 | Forward: ATACCCAGTGTCTGGAGGGTCTG  Reverse: TTTGAGCAGCTTTGCCCGCTT | 2371 | 5 |
| Exon 1B  Exon 7 | Forward: ATACCCAGTGTCTGGAGGGTCTG  Reverse: TCCAGGCGCTTTCCTGGGGA | 2595/2988 | 5 |
| Exon 1B  Exon 8 | Forward: ATACCCAGTGTCTGGAGGGTCTG  Reverse: CTCCTGCTCGCTCCTCGCA | 2641/3034 | 5 |
| Exon 1C  Exon 2 | Forward: GGGTCCAGCCCTGTATGTGGA  Reverse: GCCGCTTGAGCAGCCAGTAG | 1876 | 3 |
| Exon 1C  Exon 4 | Forward: GGGTCCAGCCCTGTATGTGGA  Reverse: GCGGGGTCCTTGTCTTGCAG | 2174 | 3 |
| Exon 1C  Exon 6 | Forward: GGGTCCAGCCCTGTATGTGGA  Reverse: TTTGAGCAGCTTTGCCCGCTT | 2658 | 5 |
| Exon 1C  Exon 7 | Forward: GGGTCCAGCCCTGTATGTGGA  Reverse: TCCAGGCGCTTTCCTGGGGA | 2882 | 5 |
| Exon 1C  Exon 8 | Forward: GGGTCCAGCCCTGTATGTGGA  Reverse: CTCCTGCTCGCTCCTCGCA | 2928 | 5 |
