# Supplementary material for: DNA Methylation Analysis of BRD1 Promoter Regions and the Schizophrenia rs138880 Risk Allele
Source: PLoS One. 2017 Jan 17;12(1):e0170121. doi: 10.1371/journal.pone.0170121 (PMC5240986; doi:10.1371/journal.pone.0170121)
Supplement: S2 Table — (DOCX) [file pone.0170121.s006.docx]

**S2 Table.** **Primer sequences and expected amplicon sizes for real-time qPCR.**

| Gene name | Primer sequence (5’>3’) | Amplicon size (bp) | Spanning  Intron |
| --- | --- | --- | --- |
| *PGK1* | Forward: AAGCTGACGCTGGACAAGCTG  Reverse: GATGCTTGGGACAGCAGCCTTA | 126 | Yes |
| *HPRT* | Forward: TGCTGAGGATTTGGAAAGGGTGT  Reverse: CCCTTGAGCACACAGAGGGCTA | 126 | Yes |
| *POLR2A* | Forward: TGCCCGAGACAAGACTGGCT  Reverse: CGCTTGCCCTCGACGTTCTG | 150 | Yes |
| *RPS13* | Forward: GCCGGATTCACCGTTTGGCT  Reverse: ATTTATGCGACCAGGGCAGAGG | 100 | Yes |
| *TBP* | Forward: CGTGCCCGAAACGCCGAATA  Reverse: AATCAGTGCCGTGGTTCGTG | 84 | Yes |
| *BRD1 Exon 11-12 (Total)* | Forward: TTGGGGAGCACATGCAGACC  Reverse: ACCAAGGGGAACCATTTTGGACT | 110 | Yes |
| *BRD1*  *Exon 1C* | Forward: GGGTCCAGCCCTGTATGTGGA  Reverse: ACCAGTCCCCTCTCGGACCA | 148 | No |
| *BRD1*  *Exon 1B* | Forward: ATACCCAGTGTCTGGAGGGTCTG  Reverse: GGGCCTGAGAAAAAGCCCTCA | 111 | No |
| *BRD1*  *Exon 1A* | Forward: CGGCGCCCCGAAGGTAATC  Reverse: AGCTTGAGCGTAGGTCAGCGT | 135 | Yes |
