# Supplementary material for: DNA Methylation Analysis of BRD1 Promoter Regions and the Schizophrenia rs138880 Risk Allele
Source: PLoS One. 2017 Jan 17;12(1):e0170121. doi: 10.1371/journal.pone.0170121 (PMC5240986; doi:10.1371/journal.pone.0170121)
Supplement: S3 Table — (DOCX) [file pone.0170121.s007.docx]

**S3 Table. Primer sequences, expected amplicon sizes, and PCR annealing temperatures for bisulfite sequencing and pyrosequencing.**

| Assay name | Primer sequence (5’>3’) | Amplicon size (bp) | Annealing  Temp. |
| --- | --- | --- | --- |
| *Bisulfite seq. Region 1* | Forward: GTATTATTTTTGTTGGGGTAT  Reverse: AAACCTCCTATATACTACAAA | 437 | 54 |
| *Bisulfite seq. Region 2* | Forward: GGTTTAGAGTTTATAGTTGTAGGAT  Reverse: TATCCCCTCAAACCAATACAA | 349 | 58 |
| *Bisulfite seq. Region 3* | Forward: TAGAGGTGATATTATAGTTTGT  Reverse: ATTCAATTCTCCCCTTATAA | 245 | 52 |
| *Bisulfite seq. Region 4* | Forward: GGGGTTTAGTTGGAGGTT  Reverse: AATAACCCCTAACCTCAACAC | 601 | 59 |
| *Pyrosequencing Region 2* | Forward: GTTAGGGGTAGGAGATAGATT  Reverse: Biotin-TATCCCCTCAAACCAATACAA  Sequencing: GTTTGTTTATTTTTTATAGATAGGT | 113 | 56 |
| *Pyrosequencing Region 3* | Forward: TAGAGGTGATATTATAGTTTGT  Reverse: Biotin-ATTCAATTCTCCCCTTATAA  Sequencing 1: GTGATATTATAGTTTGTAGAGAG  Sequencing 2: TTTTTTTAGATAGTAAAGTTTTGAG | 245 | 52 |
