# Supplementary material for: DNA Methylation Analysis of BRD1 Promoter Regions and the Schizophrenia rs138880 Risk Allele
Source: PLoS One. 2017 Jan 17;12(1):e0170121. doi: 10.1371/journal.pone.0170121 (PMC5240986; doi:10.1371/journal.pone.0170121)
Supplement: S4 Table — (DOCX) [file pone.0170121.s008.docx]

**S4 Table. Summary statistics for 6 selected DNA methylation differences between fetal and postnatal human cortex and their correlation to *BRD1* expression.**

| Probe ID^1^ | Difference in methylation  (fetal versus postnatal) | | | Correlation to *BRD1* expression | | |
| --- | --- | --- | --- | --- | --- | --- |
|  | Mean Difference | *p*-value | *q*-value | *R* | *p*-value | *q*-value |
| cg15144773 | -0.012 | 1.01E-4 | 1.67E-4 | -0.221 | 4.52E-4 | 1 |
| cg21032013 | 0.006 | 0.136 | 0.163 | -0.036 | 0.566 | 1 |
| cg02550151 | 0.056 | 1.65E-19 | 7.80E-19 | 0.533 | 1.05E-05 | 4.82E+00 |
| cg15145965 | 0.051 | 1.10E-06 | 3.56E-06 | 0.509 | 8.41E-04 | 3.84E+02 |
| cg06057569 | 0.114 | 2.94E-22 | 1.50E-21 | 0.549 | 5.53E-07 | 2.53E-01 |
| cg16001335 | 0.127 | 1.11E-57 | 1.45E-56 | 0.711 | 9.86E-26 | 4.50E-20 |

1) Data extracted from Jaffe et al. Nat Neurosci. 2016;19: 40-47.
